# Genetics of Alzheimer's Disease

**DOI:** 10.1155/2013/254954

**Published:** 2013-07-25

**Authors:** Perry G. Ridge, Mark T. W. Ebbert, John S. K. Kauwe

**Affiliations:** ^1^401 WIDB, Department of Biology, Brigham Young University, Provo, UT 84602, USA; ^2^500 W. Chipeta Way, ARUP Institute for Clinical and Experimental Pathology, Salt Lake City, UT 84108, USA

## Abstract

Alzheimer's disease is the most common form of dementia and is the only top 10 cause of death in the United States that lacks disease-altering treatments. It is a complex disorder with environmental and genetic components. There are two major types of Alzheimer's disease, early onset and the more common late onset. The genetics of early-onset Alzheimer's disease are largely understood with variants in three different genes leading to disease. In contrast, while several common alleles associated with late-onset Alzheimer's disease, including APOE, have been identified using association studies, the genetics of late-onset Alzheimer's disease are not fully understood. Here we review the known genetics of early- and late-onset Alzheimer's disease.

## 1. Introduction

Alzheimer's disease (AD) is a devastating disease characterized by decreased cognition and is also the most common form of dementia affecting an estimated 24 to 35 million people worldwide [1–3]. Incidence is further expected to increase to 1 in 85 people by 2050 because of an aging population [2]. Persons diagnosed with AD typically survive 3 to 9 years after diagnosis [1]. Full-time care is often required as AD progresses, further impacting patients and their loved ones. With the anticipated increase in AD incidence, it is essential to achieve early diagnosis, effective treatments, and a better understanding of the underlying etiology.

Effective AD diagnostics remain elusive given the disease's similarity to other dementias and poorly understood etiology. The National Institute of Neurological and Communicative Disorders and Stroke and the Alzheimer's disease and Related Disorders Association have jointly established criteria for AD diagnosis [4]. A diagnosis of probable AD is made based on meeting criteria in two areas: (1) core diagnostic criteria; and (2) supportive features. To receive a diagnosis of probable AD, a person must meet all criteria for core diagnostic criteria and one of four possible supportive features. Certain exclusion criteria exist, which if present, prevent diagnosis of probable AD. Diagnosis based on the core criteria is challenging because the criteria rely primarily on clinical observations and history. A full description of AD diagnosis can be found in Dubois et al. [4]. These are new criteria and are still used primarily for research in some countries.

Understanding AD etiology will be critical to effectively diagnose and treat the disease; however, while a number of hypotheses exist, the exact cause of AD is unknown. The most widely accepted hypothesis is the amyloid cascade hypothesis [5]. Amyloid precursor protein (APP) is cleaved by two pathways. In the nonamyloidogenic pathway, full length APP is cleaved by *α* and *γ*-secretases to produce a secreted C-terminal fragment of 83 residues. Cleavage via the *β* and *γ*-secretases can be promiscuous and produces several species of amyloid beta (A*β*) fragments. The most common fragment consists of 40 residues (A*β*_40_) and is known to inhibit amyloid deposition [6]. A fragment consisting of 42 residues (A*β*_42_) is also commonly produced. A*β*_42_ self-aggregates and can grow into extracellular fibrils arranged into *β*-pleated sheets which are the insoluble fibers of neuritic and diffuse plaques (NPs) [1]. This is thought to be the first step in AD development [7]. Subsequently, intracellular neurofibrillary tangles (NFTs) are formed, which are largely composed of hyperphosphorylated tau proteins. The formation of NFTs is largely thought to be driven by the accumulation of NPs [1]. The presence of NPs and NFTs is the hallmark pathologies of AD [8].

Another hypothesis of AD involves the mitochondria. It is widely accepted that mitochondrial function is disrupted in the brains of AD patients [1, 9–13] and that NPs aggregate within mitochondria [14, 15]. It is not known, however, whether mitochondrial dysfunction is a cause or effect of NP aggregation [11]. These questions led to the proposal of the mitochondrial cascade hypothesis [10]. Briefly, mitochondrial function and morphology change and decline with age [13, 16]. As function begins to decline, mitochondria try to compensate. During this phase, the compensation causes alterations in the mitochondria. Finally, as the mitochondria begin to fail, there are additional compensatory changes. Changes such as A*β* aggregation and tau phosphorylation are some of the transformations that occur as a result of compensating and failing mitochondria; however, the mitochondrial cascade, if correct, likely only explains a subset of AD cases. In contrast to the mitochondrial cascade hypothesis, in the amyloid cascade hypothesis, changes such as A*β* aggregation and tau phosphorylation happen first and lead to the dysfunction of mitochondria [10, 13, 17]. Each of these hypotheses is likely to be affected by both genetic and nongenetic factors.

Various nongenetic factors impact both risk for and protection from AD—the greatest of which is age [1, 18]. Other risk factors include hypertension, estrogen supplements [19], smoking [20, 21], stroke, heart disease, depression, arthritis, and diabetes [22], although some of these may be early signs of disease rather than risk factors. On the other hand, certain lifestyle choices appear to decrease the risk of AD: exercise [23], intellectual stimulation [24], and maintaining a Mediterranean diet (including fish) [25, 26]. While these nongenetic factors may affect AD risk, genetics play a critical role. The genetics of AD are complicated, however, as it is a highly heterogeneous disorder.

Several genes are known to harbor either causative or risk variants for AD. There are two primary types of AD as defined by age. The first is early-onset AD (EOAD), and the second type is late-onset AD (LOAD). Each has a unique set of causative or risk modifying genetic factors. EOAD genes are known to harbor mutations that cause AD. In contrast, LOAD genes are associated with risk for AD, but known alleles are insufficient to cause AD. In this review, we will discuss the genetics of AD, including a discussion of causative genes as well as genes with replicable association with AD.

## 2. Genetics

### 2.1. Early-Onset Alzheimer's Disease

Early-onset AD begins before age 65, and incidence estimates range from 0-1% [27] to 6%-7% [19] of total AD cases. While EOAD is believed to be dominantly inherited, it is not fully penetrant. In fact, fewer than 13% of EOAD cases demonstrate a fully penetrant autosomal dominant inheritance for multiple generations [19]. Mutations in three different genes are known to cause EOAD: amyloid beta (A4) precursor protein (APP) [28], presenilin 1 (PSEN1) [29], and presenilin 2 (PSEN2) [30]. The majority of these mutations appear to be dominantly inherited; however, not all are completely penetrant. Clinical features and pathology vary depending on the mutation's locus and position within each gene.

#### 2.1.1. APP

APP is located on chromosome 21 (21q21.2-21q21.3) and was one of the first causal genes identified for AD. There are at least 10 different APP isoforms. The primary transcript (NM_000484, NP_000475) is also the longest transcript with 18 exons. The exact function of APP is not certain, but several possible functions have been suggested such as synaptic development [31], neuronal migration [32], or as a receptor, although there have been arguments against this [33]. It is clear, however, that APP is cleaved into A*β* molecules, including A*β*_42_, which are secreted and can then accumulate in the brain forming NPs [1]. At least 25 pathogenic mutations have been identified in APP with the majority located in or adjacent to the A*β* domain (http://www.molgen.ua.ac.be/ADMutations) [33, 34]. Duplications of APP, including in Down's syndrome patients [35], are sufficient in many cases to cause EOAD due to increased A*β*_42_ production and deposition [36, 37]. Mutations in APP account for 13–16% of all EOAD cases [38, 39].

There is substantial phenotypic heterogeneity in individuals with EOAD resulting from sequence variation in APP depending on exactly where the variant is located in the gene. Mutations are typically grouped into before, in, and after the A*β* domain [40]. Depending on the mutation, A*β*_42_ levels may increase, A*β*_42_ and A*β*_40_ levels may increase (as in the case of the Swedish mutation), or total A*β* production may decrease [41–44]. The Swedish, Arctic, and London mutations are three prominent APP variants [28, 44–48]. These mutations are located in different domains of APP and lead to EOAD by different mechanisms. The Arctic mutation (E693G, inside the A*β* domain) appears to be dominantly inherited and fully penetrant with an average age of onset of 57 years and results in lower total A*β*_42_ and A*β*_40_ levels with ratios similar to wild type and leads to protofibril formation [44, 47]. In contrast to the Arctic mutation, the Swedish and London mutations flank the A*β* domain. The Swedish mutation is actually a double mutation before the A*β* domain (K670 M and N671 K) resulting in increased total A*β* production and changes inintercellular A*β* localization [45]. Finally, the London mutation (V717I) is located after the A*β* domain and results in higher A*β*_42_ [28].

#### 2.1.2. PSEN1

PSEN1 is located on chromosome 14 (14q24.3) and has at least two isoforms. Of the three genes known to cause EOAD, mutations in PSEN1 account for a greater percentage of EOAD cases (18–50%) than either of the other genes [49–51]. To date, there are at least 185 known AD causing mutations in PSEN1 (http://www.molgen.ua.ac.be/ADMutations) [34, 52]. PSEN1 EOAD is autosomal dominant; however it is incompletely penetrant. Furthermore, there can be substantial variation in age at onset (mean 45.5 years old), rate of progression, and severity of disease (average survival after diagnosis 8.4 years) [53]. Some of the variation is attributed to specific mutations in PSEN1 [54–56]. PSEN1 is a component of *γ*-secretase, which is one of the secretases responsible for APP cleavage [57]. Mutations in PSEN1 can change the secretase activity of *γ*-secretase and increase the ratio of A*β*_42_ to A*β*_40_-and A*β*_42_ more readily forms NPs [58, 59]. In general, PSEN1 mutations can be grouped into two groups: before protein position 200 and after. Pathology resulting from mutations before position 200 resembles the pathology found in sporadic AD cases, whereas mutations at subsequent positions in the protein result in more severe amyloid angiopathy [60].

#### 2.1.3. PSEN2

PSEN2 is located on chromosome 1 (1q31-q42) and has two known isoforms. EOAD causing mutations in PSEN2 are relatively rare compared to PSEN1, have higher age of onset (53.7 years old), live longer after diagnosis (10.6), appear to have a more variable penetrance, and have not been as extensively studied [53, 61]. To date, there are 12 known pathogenic mutations in PSEN2 [34, 52]. While the exact function of PSEN2 is unknown, it is believed to have a similar function to PSEN1 (as described before) [62] and to cause AD pathology by increasing A*β*_42_ levels [57].

### 2.2. Late-Onset Alzheimer's Disease

The second type of AD is late-onset AD (LOAD) or sporadic AD. Even though numerous genetic risk factors and biomarkers have been identified for LOAD, no causative gene has been identified. While there are many genes associated with LOAD, ten different loci (Table 1) meet all the criteria to be included in the “Top Results” list of the Alzheimer Research Forum or ALZGENE (accessed October 2011, for details about construction of the list see http://www.alzgene.org/) for associations with AD [63]. In this section we briefly introduce each of these loci in the following groups (grouped by common function, pathway, or family): apolipoproteins and lipid homeostasis, genes involved in endocytosis, MS4 family proteins, and other loci. We also review recently identified rare AD variants.

#### 2.2.1. Apolipoproteins and Lipid Homeostasis

Apolipoproteins are a family of proteins involved in lipid homeostasis. These proteins bind and transport lipids through the lymphatic and circulatory systems. Two different apolipoproteins and an ABC transporter have been shown to associate with AD. The first is apolipoprotein E (APOE), which is located on chromosome 19 (19q13.2) and consists of four total exons (three coding). There is only one major isoform (NM_000041, NP_000032), which encodes protein 317 amino acids in length. APOE is a component of the chylomicron and plays a pivotal role in very low density lipoprotein clearance from circulation [64]. Impaired function of APOE results in increased plasma levels of cholesterol and triglycerides [64].

There are three primary APOE alleles: *ε*2 (rs429358), *ε*3 (wild type), and *ε*4 (rs7412). These alleles differ by substitutions at positions 112 and 158 (protein positions correspond to the processed protein) where the wild type allele *ε*3 is Cys112 and Arg158, *ε*2 is Cys112 and Arg158Cys, and *ε*4 is Cys112Arg and Arg158. *ε*3 has an estimated population frequency of 78.3% (8.5%–98%), whereas *ε*2 has a population frequency of 6.4% (0%–37.5%) and *ε*4 14.5% (0%–49%) [65]. The *ε*4 allele is the risk allele and is the most significant known genetic risk factor for LOAD. This allele was first identified as a genetic risk factor for LOAD in 1993 by Corder et al. [66]. The association for this allele has been replicated numerous times in various ethnic groups including Caucasians [66], African Americans [67, 68], Asians [69, 70], and Hispanics [68]. The *ε*4 allele is the only widely accepted genetic risk factor for LOAD [71] and increases risk with increasing *ε*4 dosage. In contrast, *ε*2 decreases AD risk [72]. Possible APOE genotypes, listed in order of AD risk, are *ε*2/*ε*2, *ε*2/*ε*3, *ε*3/*ε*3 or *ε*2/*ε*4, *ε*3/*ε*4, and *ε*4/*ε*4 [72]. Although AD risk is much higher in persons with one or more *ε*4 alleles, *ε*4 is not causative and some individuals homozygous for *ε*4 never develop AD [66].

Despite APOE's importance in AD genetics, its exact role in AD is unknown. Levels of A*β*_42_ deposition in the brain are, however, correlated with the number of *ε*4 alleles [73], and APOE is hypothesized to be involved in the clearance of A*β*_42_ from the brain, proteolytic degradation of A*β*_42_, and astrocyte mediated degradation of A*β*_42_ [74–76].

The second apolipoprotein associated with AD is clusterin (CLU). A single variant, rs11136000, in CLU has been associated with AD in multiple different ethnic groups as a protective allele [71, 77–90] and has been associated with lower levels of A*β*_42_ [91]. CLU, also known as apolipoprotein J, is located on chromosome 8 (8p21-p12). It has been suggested that CLU may increase the toxicity of A*β*_42_ [92] and that it is involved in A*β*_42_ clearance [93, 94]. Additionally, AD affected people have increased CLU in circulation, and CLU levels are correlated with a higher rate of cognitive decline [95–97]. Lastly, A*β* increases CLU expression [98], and there may be a direct interaction between A*β*_40_ and CLU [99–101].

Another gene, ATP-binding cassette, subfamily A (ABC1), member 7 (ABCA7), was recently identified as an AD susceptibility locus based on a significant association between rs3764650 and AD [86, 102], where rs3764650 is located in intron 13 of ABCA7. ABCA7 is an ATP-binding cassette transporter used to move numerous molecules across membranes, and interference of ABCA7 decreases phagocytosis [103]. ABCA7 helps maintain lipid homeostasis through its role in lipid transport across the cellular membrane [104, 105]. Additionally, ABCA7 expression is responsive to lipoprotein levels and type [106]. Lipid dysfunction, changes in lipid homeostasis, and modifications of neuronal membrane homeostasis can all cause numerous diseases, including AD [107–109]. This provides a basis for how ABCA7 can lead to AD. rs3764560 is associated with increased risk for AD and, given ABCA7's role in lipid transport and phagocytosis, likely disrupts, or is in linkage disequilibrium (LD) with a variant that disrupts lipid homeostasis and/or membrane homeostasis.

#### 2.2.2. Genes Involved in Endocytosis

Other important groups of genes are genes involved in endocytosis. Endocytosis is the process a cell uses to transport molecules across the cell membrane into the cell. Previous studies have demonstrated a role for endocytosis in AD generally, and clathrin-mediated endocytosis specifically [110]. Generally, APP is processed in endosomes; therefore endocytosis of APP from the cell surface is necessary for A*β*_42_ production, while specifically inhibiting clathrin-mediated endocytosis decreases levels of A*β*_42_ [110]. As such, endocytosis is a primary interest in AD etiology, and several genes involved in endocytosis such as BIN1, PICALM, CR1, and CD2AP are, unsurprisingly, associated with AD.

The first of these, bridging integrator 1 (BIN1), is located immediately downstream of rs744373, an SNP associated with AD [71, 77, 82, 86, 87, 90, 102, 111]. BIN1 is located on chromosome 2 (2q14) and has at least 10 different isoforms. BIN1 has multiple functions. First, BIN1 is involved in synaptic vesicle endocytosis [87, 112]. Like clathrin-mediated endocytosis, although to a lesser extent, synaptic activity endocytosis has a role in APP processing [110]. Second, BIN1 decreases the formation of clathrin-coated vesicles—a necessary step in clathrin-mediated endocytosis [113]. Mutations in BIN1 could, hypothetically, have different effects on the risk for AD. Variants that adversely affect BIN1's role in synaptic vesicle endocytosis would likely be protective since they would decrease APP processing efficiency. In contrast, variants that prevent BIN1 from inhibiting clathrin-coated vesicle formation would increase clathrin-mediated endocytosis and APP processing, resulting in increased A*β*_42_ production. These variants would increase risk for AD. A single variant could conceivably have both effects; however, since clathrin-mediated endocytosis has a larger role in APP processing, the net effect would increase AD risk. rs744373 in BIN1 is one potential example and is associated with increased AD risk.

Another gene associated with AD and endocytosis is phosphatidylinositol binding clathrin assembly protein (PICALM) located on chromosome 11 (11q14) and has at least four known isoforms. Harold et al. [71] identified a single variant, rs3851179, associated with increased AD risk. This same association has been replicated several times [78, 82, 83, 86, 87, 114]. PICALM is involved in protein trafficking and synaptic vesicle endocytosis and may control levels of GluR2 and VAMP2 [112, 115, 116]. Its main function, however, is as a clathrin assembly protein, where it increases clathrin-coated vesicle assembly and helps regulate the amount of membrane recycling and clathrin-mediated endocytosis [115, 117]. The finding that rs3851179 is a protective allele against AD is consistent with a hypothesis that this variant decreases formation of clathrin-coated vesicles by disrupting PICALM function.

Another gene in the endocytic set associated with AD is complement component (3b/4b) receptor 1 (CR1). CR1 was first identified as a risk locus for AD in 2009 (rs3818361) [71, 85, 114], with replication in several ethnic groups [78, 79, 83, 87, 118]. CR1 is located on chromosome 1 (1q32) and has at least two known isoforms. Although an exact function for CR1 is not known, it has been suggested that CR1, working with C3b (a complement fragment in the complement cascade), plays a role in A*β* clearance [85, 118, 119]. Additionally, CR1 appears to facilitate endocytosis [120]. rs3818361 is associated with increased risk for AD. Variants in CR1 could potentially cause AD by disrupting its A*β* clearing function or by a gain-of-function mutation resulting in increased endocytosis.

Lastly, rs9349407 in a new AD susceptibility gene named CD2-associated protein (CD2AP) was recently reported [86, 102]. CD2AP is located on chromosome 6 (6p12) and is responsible for regulation of the actin cytoskeleton [121, 122]. CD2AP is additionally involved in receptor-mediated endocytosis [123]. Changing endocytosis can modify lipid homeostasis and APP processing, among other things, and is a plausible explanation for how rs9349407, or a variant in LD with rs9349407, could cause AD.

#### 2.2.3. MS4A6A and MS4A4E

MS4 family proteins are another essential gene set in AD. Membrane-spanning 4 domains, subfamily A, member 6A (MS4A6A) and membrane-spanning 4 domains, subfamily A, member 4E (MS4A4E) were recently identified as AD risk loci with rs610932 (MS4A6A) and rs670139 (MS4A4E) showing association with AD [71, 86, 102]. rs610932 is located in the 3′-UTR of MS4A6A, and rs670139 is in the intergenic region between MS4A6A and MS4A4E. Each has a different association with AD where rs610932 is protective and rs670139 increases AD risk. MS4A6A and MS4A4E are located together on chromosome 11 (11q12.1 and 11q12.2, resp.) with at least four and one known isoform(s), respectively, and are located in a cluster with other MS4A (membrane-spanning 4 domains subfamily A) subfamily genes [124, 125]. Very little is known about the function of either of these genes.

#### 2.2.4. Other

Another locus associated with AD, which did not fit in any of the previous categories is rs3865444 in CD33 molecule (CD33). An association for rs3865444 was initially identified in 2008 [126] and was subsequently replicated several times [71, 86, 102, 127]. CD33 is a myeloid antigen located on chromosome 19q13.3 with at least three known isoforms and is expressed in a variety of tissues and cell types. Interestingly, CD33 plays a major role in leukemia [128], but no widely accepted hypotheses currently exist for its involvement in AD.

#### 2.2.5. Rare Variants (TREM2 and APP)

In addition to loci reported on the Alzheimer Research Forum and ALZGENE, several groups recently identified two rare variants using novel study designs by combining next-generation sequencing and AD genetics. The first, rs63750847, is located in APP [129]. This missense variant is extremely rare (estimated frequency of 0.038%) and observed almost exclusively in people of Icelandic descent. This variant seems to confer protection against AD (odds ratio of 5 to 7 depending on the control group). In contrast, APOE *ε*4, the largest known risk variant, has an odds ratio of 3.7. This variant is located close to the BACE1 cleavage site and results in reduced A*β*_42_ production [129]. Interestingly, elderly controls bearing rs63750847 also experienced less cognitive decline than noncarrier controls suggesting shared physiology for both normal and AD-related cognitive decline.

A second rare variant, rs75932628, was recently identified in TREM2 [130, 131]. rs75932628 is a missense risk variant with a population frequency of 0.3% and odds ratio of ~3. This variant is hypothesized to increase risk for AD by disrupting the role of TREM2 in the regulation of phagocytosis and/or the inflammatory response [130]. We believe that these rare variants and others yet to be identified explain a large portion of genetic risk for AD. As such, a greater effort to identify any remaining variants must be a priority in AD research.

#### 2.2.6. Mitochondrial Genetics and Alzheimer's Disease

As previously explained, mitochondria malfunction in AD is well known, but it is unclear whether these changes are a cause or effect of AD. Similarly, what role, if any, the mitochondrial genome has in AD risk is unknown even though numerous studies have been performed analyzing mitochondrial variation and/or haplotypes to identify sequence features in the mitochondrial genome associated with AD. While a number of these studies have identified significant associations, there is no consensus and some of these studies offer conflicting results. In Table 2, we list a summary of studies looking at variation in the mitochondrial genome and its role in AD.

## 3. Endophenotypes of Alzheimer's Disease

The use of endophenotypes of Alzheimer's disease to understand the genetic basis for AD risk is becoming more common. Cerebrospinal fluid levels of A*β*_42_ and tau are perhaps the most accepted biomarkers for AD and have recently been used both to characterize the biological effects of known risk factors and to identify novel AD risk markers. Using quantitative endophenotypes instead of qualitative case/control status as the phenotype for a genetic study may reduce heterogeneity in clinical diagnosis, thus increasing power to detect genetic associations [146]. In addition, this approach can provide more specific hypotheses for the biological mechanism by which associated variants alter risk. Large-scale association studies of cerebrospinal fluid levels of *Aβ*_42_ and tau/p-tau have successfully identified variants in several genes that alter risk or rate of progression of Alzheimer's disease [147–149]. Genetic variants in PPP3R1 and MAPT have been shown to be associated with cerebrospinal fluid p-tau levels and rate of decline in Alzheimer's disease patients in three independent samples [147, 149]. The largest genome-wide association study of cerebrospinal fluid tau levels to date identified three loci that show significant association. Two of these loci do not show evidence for association with AD risk or other AD related traits. The third locus (rs9877502) is on chromosome 3 between GEMC1 and OSTN. This locus shows significant association with several Alzheimer's disease phenotypes including AD risk, neurofibrillary tangle counts, and cognitive decline.

Cerebrospinal fluid levels of A*β*_42_ and tau/p-tau have also been used to characterize the biological effects of reported Alzheimer's disease risk markers. The APOE *ε*4 allele shows strong and replicable association with cerebrospinal fluid A*β*_42_ and tau levels in several studies. Significant associations between variants in CLU, MS4A4A, and SORL1 and cerebrospinal A*β*_42_ levels [91, 150] and between variants in CLU, PICALM, and CR1 and cerebrospinal tau levels have been reported [148, 151, 152]. The recent success of these approaches to both characterize newly discovered AD risk variants and identify novel risk variants suggests that the use of endophenotypes is an important part of the ongoing effort to solve the genetic architecture of AD.

## 4. Conclusions

Here we reviewed known genetic risk and protective factors of AD. Research findings thus far are substantial; however, we still know relatively little about the genetics of AD. 11 nuclear markers have been identified by association studies, and all but one of these have a small effect on risk (the two APOE alleles have larger effect). Additionally, these are not causative variants, even the APOE alleles, but are only associated with disease status. Functional variants have not been identified for any of the known AD markers. Many of the limitations that restricted our ability to find causative and additional AD biomarkers in the past no longer exist, and it is clear that many AD variants remain unidentified [153]. These unidentified variants, like the APP and TREM2 variants, will likely be rare, have large effect on risk, and require innovative study designs to discover. The application of next-generation sequencing to AD genetics will provide the necessary information to identify additional disease variants. The sequencing of large numbers of AD cases and controls (as in the case of APP and TREM2) will reveal additional, large effect AD variants, and the sequencing of large families will reveal rare, highly penetrant AD variants.

The study of epistasis is another area likely to add to our understanding of the genetics of AD, and recently, many researchers have called for an increased focus and developing more robust approaches to study gene-by-gene interactions [154–161]. Preliminary research has yielded a number of discoveries across diseases such as cancer, rheumatoid arthritis, and AD [162–187] and improved analytical methods [188–192]. Discovered interactions that affect AD risk include (1) IL-6 and IL-10 discovered by Infante et al. [193] and replicated by Combarros et al. [184]; (2) GSTM3 and the HHEX/IDE/KIF11 locus discovered by Bullock et al. [185]; (3) HMGCR and ABCA1 discovered by Rodríguez-Rodríguez et al. [186]; and TF and HFE first reported by Robson et al. [194] and replicated by Kauwe et al. [187].

There are, however, many challenges remaining. For instance, in 2009 Combarros et al. attempted to replicate more than 100 epistatic findings and were only able to replicate 27 [188], suggesting that many epistatic interactions may be false positives. Clearly current approaches need to be improved before we can efficiently study epistasis.

There have been huge advances in our understanding of the genetics of AD over the last few years. These advances are promising and illustrate the power and utility of modern approaches. As we begin to leverage datasets with increasing number of individuals and complete genomic coverage, we will have the opportunity to unravel the complexities of the genetic architecture of this disease, including the effects of rare variants and epistasis. This information provides the foundation for the development of preventative and curative therapies.

## Figures and Tables

**Table 1:** Late-onset Alzheimer's disease associated genes/variants.

Variant	Gene	Abbreviation	Risk/protective
rs7412	Apolipoprotein E	APOE	Risk
rs429358	Apolipoprotein E	APOE	Protective
rs744373	Bridging integrator 1	BIN1	Risk
rs11136000	Clusterin	CLU	Protective
rs3764650	ATP-binding cassette, subfamily A (ABC1), member 7	ABCA7	Risk
rs3818361	Complement component (3b/4b) receptor 1 (Knops blood group)	CR1	Risk
rs3851179	Phosphatidylinositol binding clathrin assembly protein	PICALM	Protective
rs610932	Membrane-spanning 4 domains, subfamily A, member 6A	MS4A6A	Protective
rs3865444	CD33 molecule	CD33	Protective
rs670139	Membrane-spanning 4 domains, subfamily A, member 4E	MS4A4E	Risk
rs9349407	CD2-associated protein	CD2AP	Risk

Each of the top variants associated with late-onset Alzheimer's disease from meta-analysis done by the Alzheimer Research Forum is listed here, together with the specific associated variant, and whether the variant increases risk or provides protection.

**Table 2:** Mitochondrial variation/haplogroups associated with AD.

Haplogroup	Dataset	Effect	Ethnicity	No. cases/controls
B4C1 [132]	Selected SNPs	Risk	Japanese	96/384
G2A [132]	Selected SNPs	Risk	Japanese	96/384
HV [133]	Haplogroups, SNPs	Risk	Polish	222/252
H [134]	HVS-I sequence	Risk	Iranian	30/100
H5/H5A [135]	D-loop sequence, restriction analysis	Risk	Italian	936/776
H6A1A/H6A1B [136]	Full mtDNA sequences	Protective	Caucasian	101/632
K [137]	Haplogroups	Protective	Italian	N/A*
N9B1 [132]	Selected SNPs	Risk	Japanese	96/384
U [134, 138]	HVS-I sequence, 10 SNPs	Risk	Iranian, Caucasian	30/100, 989/328**
U [137, 138]	Haplogroups, 10 SNPs	Protective	Italian, Caucasian	N/A*, 989/328**
UK [139]	138 SNPs	Risk	Caucasian	170/188
None [140]	4 SNPs	None	Unknown	70/80
None [141]	European haplogroups	None	Unknown	185/179
None [142]	U, K, J, and T haplogroups	None	English	185/447
None [143]	European haplogroups	None	Tuscan	209/191
None [144]	Haplogroups	None	Finnish	128/99***
None [145]	138 SNPs	None	Caucasian	3250/1221

*The authors showed that haplogroups U and K neutralized the risk of the APOE e4 allele.

**The authors demonstrated an increased risk for AD for males with haplogroup U and decreased risk for females with haplogroup U.

***These were early onset AD cases.
